# Incidence of Tuberculosis in Inflammatory Rheumatic Diseases: Results from a Lithuanian Retrospective Cohort Study

**DOI:** 10.3390/medicina56080392

**Published:** 2020-08-05

**Authors:** Dalia Miltinienė, Giedrė Deresevičienė, Birutė Nakčerienė, Valerija Edita Davidavičienė, Edvardas Danila, Irena Butrimienė, Jolanta Dadonienė

**Affiliations:** 1 State Research Institute Centre for Innovative Medicine, LT-08406 Vilnius, Lithuania; jolanta.dadoniene@mf.vu.lt; 2 Institute of Clinical Medicine, Clinic of Rheumatology, Orthopaedics Traumatology and Reconstructive Surgery, Vilnius University Faculty of Medicine, LT-03101 Vilnius, Lithuania; 3 Centre of Rheumatology, Vilnius University Hospital Santaros Klinikos, LT-08661 Vilnius, Lithuania; giedre.dereseviciene@santa.lt (G.D.); irena.butrimiene@santa.lt (I.B.); 4 Programs and Tuberculosis State Information System Department, Vilnius University Hospital Santaros Klinikos, LT-08661 Vilnius, Lithuania; bnakceriene@gmail.com(B.N.); edita.davidaviciene@santa.lt(V.E.D.); 5 Vilnius University Life Sciences Center, LT-10257 Vilnius, Lithuania; 6 Centre of Pulmonology and Allergology, Vilnius University Hospital Santaros Klinikos, LT-08661 Vilnius, Lithuania; edvardas.danila@santa.lt; 7 Institute of Clinical Medicine, Clinic of Chest Diseases, Immunology and Allergology, Vilnius University Faculty of Medicine, LT-03101 Vilnius, Lithuania; 8 Institute of Health Sciences, Department of Public Health, Vilnius University Faculty of Medicine, LT-03101 Vilnius, Lithuania

**Keywords:** rheumatic diseases, tuberculosis, incidence

## Abstract

*Background and objective:* With an increase in survival rates among rheumatic patients, comorbidities and infections, in particular, have gained more importance, especially after the introduction of biologicals to the treatment algorithms. Tuberculosis (TB) infection has always been given a special attention in patients with rheumatic diseases (RD). Although Lithuanian population has one of the highest TB incidence rates among European countries, the incidence of TB in the rheumatic patients’ population is still unknown. The aim of this study was to assess the incidence rate of TB in an inflammatory RD retrospective cohort and to compare that rate with a rate in a general population. *Material and Methods:* Patients with the first-time diagnosis of inflammatory RD during the period between 1 January 2012 and 31 December 2017 were identified from the Lithuanian Compulsory Health Insurance Information System database SVEIDRA. All cases were cross-checked with Health Information center at the Institute of Hygiene, for the vital status of these patients and date of death if the fact of death was documented, and with Tuberculosis Register operated by Vilnius University Hospital Santaros Klinikos, for the confirmation of TB cases. Sex and age standardized incidence ratios (SIR) were calculated by dividing the observed numbers of TB among rheumatic patients by the expected number of cases, calculated using national rates from Lithuanian Department of Statistics Official Statistics website. *Results:* Overall, 8779 patients with newly diagnosed RD were identified during the 2013–2017 period, these included 458 patients who used biological disease modifying drugs (bDMARDs). The mean duration of the follow-up period was 2.71 years. The cohort consisted mainly of women (70%) and a half of the cohort were rheumatoid arthritis (RA) patients (53%). Mean age of patients at the time of RD diagnosis was 56 years (range = 18–97 years). There were 9 TB cases identified during 23,800 person years of follow-up: 2 cases among them were treated with bDMARDs. The mean calculated annual TB incidence in RD cohort was 37.81 per 100,000 person years, which is consistent with the incidence rate predicted by national estimates, with a resultant SIR of 0.90 (0.41–1.70). The unadjusted hazard ratio for bDMARD use versus no bDMARD use was 4.54 (0.94; 21.87) in a total cohort and very similar in rheumatoid arthritis cohort; in both cohorts, it was not a statistically significant risk. *Conclusions:* Here, we present the first nationwide cohort study to assess the incidence of TB in a broad spectrum of inflammatory RD. Although limited by short follow-up period, this study shows that TB incidence in RD cohort does not exceed TB incidence in the general Lithuanian population.

## 1. Introduction

With an increase in survival rates among rheumatic patients, comorbidities and infections, in particular, have gained more importance, especially after the introduction of biologicals to the treatment algorithms. It is generally accepted that these patients are susceptible to several types of infections owing to suppression of their immune system caused by treatment or by the disease itself [1,2,3]. Tuberculosis (TB) infection has always been given a special attention in patients with rheumatic diseases (RD) [4,5,6,7,8]. With the introduction of TNF antagonists to the treatment of RD in 2000 in Lithuania, TB in this setting gained additional attention both because of immune system challenge presented by the disease and because Lithuanian population has one of the highest TB incidence rates among European countries [9]. Although the number of TB cases has been falling in Lithuania and globally [10], Lithuania is still considered a high-TB-incidence country: in 2018, the average TB incidence rate in the European region was 30 per 100,000 individuals [9] and in Lithuania, it was 44 per 100,000, which is the highest among the Baltic countries and one of the highest in Europe [10]. The incidence of TB in the rheumatic patients’ population in Lithuania has not been measured.

The aim of our study was to assess the incidence rate of TB in an inflammatory RD retrospective cohort and compare that rate with the general population rates. As a secondary aim, we have also assessed the incidence of TB in patients with RD treated with biological disease modifying drugs (bDMARDs).

## 2. Materials and Methods

### Data Sources

After obtaining approval from Vilnius Regional Bioethics Committee (approval number 158200-17-958-462, 7 November 2017), we have performed this study using the data of Lithuanian Compulsory Health Insurance Information System database SVEIDRA. SVEIDRA is a population-based database with data starting from 1995, although computerized data eligible for research are only available from 2005. The data captures all physician visits, procedures, hospitalizations, diagnoses, and prescribed reimbursed medications to all residents of Lithuania. The information sources are health care institutions and medication prescriptions released by pharmacies.

We have requested the information from SVEIDRA on all patients who had a first-time diagnosis of inflammatory RD during the period between 1 January 2012 and 31 December 2017. These rheumatologic conditions included rheumatoid arthritis (RA) (diagnosis codes M05 and M06 according to International Classification of Diseases 10th version (ICD-10), psoriatic arthritis (PsA) (M07), systemic connective tissue diseases (CTD) and vasculitis (M30-35), ankylosing spondylitis and spondyloarthropathies (SpA) (M45 and M46). We have also requested information on the diagnosis (ICD-10 code A15-19) and date of diagnosis of TB, information about prescription of glucocorticoids (prednisolone or methylprednisolone), conventional synthetic (cs) DMARDs (methotrexate, azathioprine, leflunomide, sulfasalazine, and hydrochloroquine), or bDMARDs (infliximab, etanercept, adalimumab, tocilizumab, or rituximab with available biosimilars).

In total, 75,846 RD cases, first time diagnosed between 2012 and 2017, were selected. In order to exclude prevalent cases, at least 1 year of no RD recorded data prior to the index date was required. We excluded 22,526 cases primary diagnosed in 2012 from the total cohort as it was not possible to verify their RD diagnosis prior to 2012 because no data preceding that year was available.

**Figure 1:**
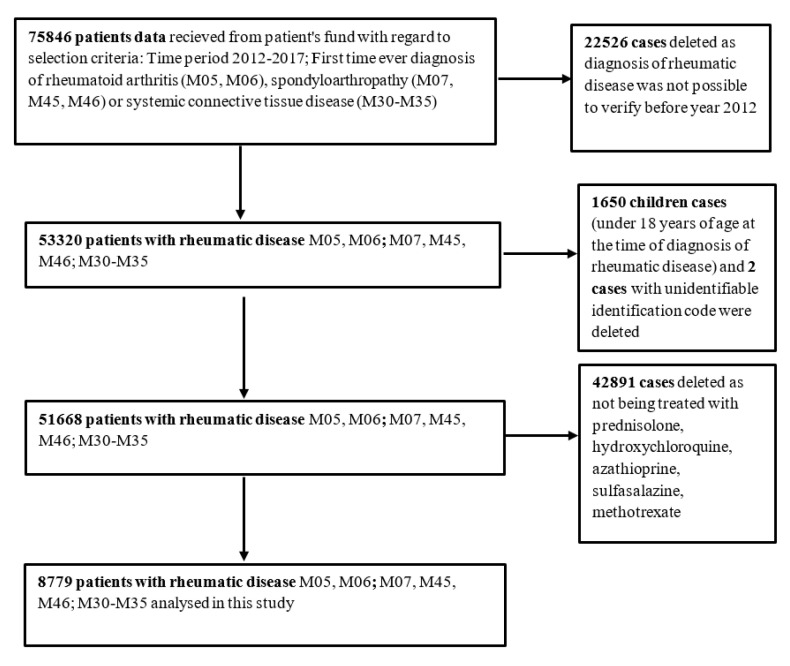
Details of study methodology.

Altogether, 1650 cases of children (<18 years old at the time of diagnosis) were deleted, as well as 2 cases with unidentifiable identification code. Participants were classified as cases if they had records of at least one prescription of the medications for RD reimbursed by the state. In particular, 42,891 cases with no information about prescribed reimbursed treatment with glucocorticoids, csDMARDs (methotrexate, azathioprine, leflunomide, sulfasalazine, and hydrochloroquine), or bDMARDs were excluded. Finally, 8779 cases were included in final analysis as is demonstrated in Figure 1.

The final 8779 cases were cross-checked with Health Information center at the Institute of Hygiene, for the vital status and date of death if the fact of death was documented, and with Tuberculosis Register operated by Vilnius University Hospital Santaros Klinikos, for the confirmation on TB cases. The patient was considered as TB case when the diagnosis was confirmed bacteriologically or by radiological or histological evidence of TB. The personal identification code was used for cross-checking the cases.

Available data for the final analysis included sex, age, ICD-10 code of RD, date of diagnosis of RD, ICD-10 code of TB diagnosis, date of diagnosis of TB, date of death, and information about the state’s reimbursement for the prescribed drugs.

For the comparison with national estimates, we have calculated the mean annual incidence rate of TB in general population of Lithuania in years 2013–2017. For this purpose, the information on new cases of all adults diagnosed with TB in the years 2013–2017 was obtained from Tuberculosis Register operated by Vilnius University Hospital Santaros Klinikos and the information on adult Lithuanian population census in 2013–2017 was obtained from Statistics Lithuania (www.stat.gov.lt) [11].

## 3. Statistical Methods

The incidence of TB in the study population was assessed using a retrospective cohort study. Person years of follow-up were calculated from the date of RD diagnosis to the first date of one of the following events: the date of TB diagnosis, death, or the end of follow-up (31 December 2017). Sex- and age-standardized incidence ratios (SIRs) were calculated by dividing the observed numbers of TB cases among rheumatic patients by the expected number of cases; the latter was calculated using national rates from Lithuanian Department of Statistics Official Statistics Website [11]; 95% confidence intervals (CIs) for SIRs were calculated as well. We used Cox proportional Hazard ratio (HR) model for TB risk assessment for bDMARDs users versus no bDMARDs users. We calculated unadjusted HR and adjusted for possible confounders, namely, age and gender. We estimated HR in the total cohort of RD patients and in the subgroup of rheumatoid arthritis. Analyses were performed using IBM SPSS Statistics for Windows, version 26 (IBM Corp., Armonk, NY, USA).

## 4. Results

During the period between 2013 and 2017, we have identified 8779 patients with RD (4623 patients with RA, 2479 with SpA (including PsA), and 1677 with systemic CTD and vasculitis), including 458 bDMARDs users (Table 1). The mean duration of follow-up was 2.71 years. The cohort consisted mainly of women (70%) and a half of the cohort were RA patients (53%). Mean age of the patients at the time of RD diagnosis was 56 years (range = 18–97 years).

In total, there were 9 TB cases identified during 23,800 person years of follow-up: 2 cases in patients treated with bDMARDs and 7 cases among those not treated with bDMARDs. TB cases were among a younger cohort of patients with the mean age of 44 years (range = 18–66 years); 5 cases were females.

## 5. Tuberculosis Cases in RD Cohort

Nine cases of TB identified in RD cohort yielded the TB incidence of 37.81 per 100,000 person years. According to data obtained from official Statistics Lithuania (www.stat.gov.lt) website [11], the TB incidence rate in adult general population of Lithuania during 2013–2017 was calculated to be 49.22 per 100,000 inhabitants. Table 2 shows the incident TB rates and corresponding SIRs compared with age- and sex-adjusted national estimates. The incidence of TB in all patients’ cohort and in separate diseases cohorts was highly consistent with that predicted by national estimates, with a resultant SIR of 0.90 (0.41–1.70). SIR in female group tend to be higher than the population estimates but not statistically significant.

All 9 identified cases of TB were newly diagnosed pulmonary TB cases. Eight of them were confirmed by culture. Resistant TB was reported in 2 out of 9 cases with the relevant drug susceptibility testing results. Both resistant cases were monoresistant (one had resistance to isoniazid and the second to pyrazinamide). Of all 9 TB cases, 6 were treated successfully, 1 person died due to TB, and 2 cases defaulted TB treatment. Two patients before contracting TB were treated with infliximab, whereas others were only on csDMARDs or glucocorticoids.

Table 3 shows the result from Cox proportional HR model for TB risk in bDMARDs users versus no bDMARDs. The unadjusted HR for bDMARDs use versus no bDMARDs use was 4.54 (0.94; 21.87) in a total cohort and very similar in rheumatoid arthritis cohort; in both cohorts, it was not a statistically significant risk. Adjusting for age and gender did not reveal any different trend in risk evaluation since threefold increase in the risk in both cohorts was not significant.

## 6. Discussion

Using national registries data from four official state-run sources, we have assessed patients with inflammatory rheumatic diseases in Lithuania and found no significantly increased incidence of TB in this cohort compared with the general population. Nine cases of TB identified in RD cohort yielded a mean annual TB incidence of 37.81 per 100,000 person years. Fourfold increased risk of TB in bDMARDs users versus those without the use of bDMARDs was observed, although the level of confidence does not allow to consider the result statistically significant, possibly because of a short follow-up time.

Our study is not in line with the previously published studies showing elevated incidence of TB in rheumatic patients [4,5]. These results are notable, given that Lithuania is a high TB incidence country, with 44 new TB cases per 100,000 people according to TB monitoring data in Europe in 2018 [9]. According to available research data in patients with RA, even those who have never used TNF inhibitors have a risk of TB of 2–10 times higher compared to the general population. This risk is probably associated with immunosuppression linked to the disease and the use of other medications such as corticosteroids [4,6,7,12]. Studies from Sweden and Finland (countries with a low incidence of TB) found that compared with the general population, even rheumatic patients not treated with biologicals had a fourfold increased risk of TB [6,13]. Studies suggest that the incidence of development of TB should be even more considerable in patients who live in countries with a high incidence of this infection in the general population [14,15,16]. In a nationwide retrospective register study performed in Finland, it was noted that between 1995 and 2007 the incidence of TB in adult RD patients with reimbursed DMARDs decreased from 58.8 to 30.0 per 100,000. This trend was similar to that in the general population and was explained by an improved standard of living and by strict TB control during the 20th century [13].

There are several articles stating that the development of TB is not a common complication in RD patients. Data supporting the statement were reported in studies of patients living in the United States, France, and Greece—countries with a low incidence of TB in the general population [1,8,17]. Andonopoulos et al. observed unselected patients with different systemic RDs who were treated with steroids [17], Wolf F et al. analyzed a group of RA patients [8], and a group of polymyositis and dermatomyositis patients was studied by Marie et al. [1]. All three studies concluded that the rate of TB was not increased in patients with different RD (except a group of RA patients treated with infliximab).

Apart from the already elevated risk of TB in rheumatic patients [4,5,6,7,12,18], this threat was further escalated in the era of RD treatment with biologicals, as reported by clinical trials and system meta-analyses [2,4,19]. Although more dramatic in highly endemic areas, a nonsignificantly increased risk was also suggested in low TB rate areas [20]. Studies have shown that following TNF antagonist therapy, the relative risk for activation of latent TB is increased up to 25 times, depending on the clinical setting and the TNF antagonist used [2,7,8,12,21,22,23,24]. Activation of latent tuberculosis following IL-1 receptor antagonist therapy has also been reported [25,26], but the risk of TB reactivation has not been thoroughly studied for non-TNF antagonists [27]. In 951 rheumatoid arthritis patients on biologicals in a retrospective cohort study conducted by Lim et al. (2017), the newer bDMARDs were less linked to TB infection if compared to classical TNF antagonists [28]. The authors of this study concluded that etanercept and adalimumab demonstrated similarly increased risks, while relatively low risks were seen in golimumab, tocilizumab, abatacept, and tofacitinib. In our study, two out of nine TB cases were treated with infliximab, whereas the rest seven have not received biological treatment at all.

Our findings are in accord with the results of the meta-analysis of 13 registries/cohort studies that reported a fourfold RR of TB associated with anti-TNF-α agents [29], although our result is not statistically significant mainly due to short period of follow-up and small number of identified TB cases.

Age is one of the acknowledged TB risk factors [30]. The mean age of TB incident cases in anti-TNF-α registries was reported to range from 53.1 to 70.2 years [6,7,8,31], which was higher than that in our study. It might be projected that since the mean age of our study participants was 56 years, and TB risk is higher in older age, the rate of TB in our cohort would increase in the future considering this comparably younger population.

TB is generally more prevalent among men by around 1.3- to 2.0-fold compared to women [10]. Recent cohort study reported male gender as an independent risk factor for TB [32]. However, the majority of TB cases in our study consisted of women, likely resulting from predominance of RA and women in our cohort. This predominance and low proportion of men in the study population could also add to the underestimation of TB in our study. Smoking is another key risk factor for TB development [24]. Although we had no information about smoking status of our cohort patients, we can make a hypothesis that smoking is less prevalent among women, which could also add to low incidence of TB in our cohort.

It is also interesting, that all 9 detected TB cases in our study had pulmonary TB, when it is reported that extrapulmonary forms account for almost 60% of cases and disseminated forms for 26% of patients treated with TNF inhibitors [33].

It is worth noting that starting from the beginning of bDMARDs era in Lithuania, algorithms of careful screening for latent TB before the treatment initiation were introduced into clinical practice. Generally, low numbers of TB cases in the total cohort of 8779 RD patients and only two cases among bDMARDs users may be linked to the policy of latent TB screening before the start of treatment with bDMARDs. The screening for latent TB includes X-ray of the lungs and computed tomography following it, if necessary, QuantiFERON-TB Gold test and tuberculin skin test, both. The patient can be prescribed with bDMARD only if lung reports and both tests, blood and skin, are negative [34]. This might result in a good control of this infection without increase of its incidence.

Several strengths of our study deserve to be addressed. The data used in this study were from computerized national registries that were prospectively recorded; therefore, there is no concern about biased recall of cases or exposure. All TB cases were verified in the TB register, ensuring that not a single case of TB was unverified.

Our study had several limitations. It was a prescription-based study and one of the exclusion criteria was no information about treatment with state-reimbursed medications, therefore, some cases of RD might be omitted in a case when patient’s medications are not reimbursed by the state (especially cases of spondyloarthropathies, whose treatment is poorly reimbursed by the state). Furthermore, no information about the duration of prescribed treatment and doses of medications was available; therefore, we could not evaluate the impact of different medications to the risk of TB.

However, the major limitation is the duration of the retrospective follow-up. The mean of the follow up period was 2.7 years and this might be too short to show the effect of RD itself or its treatment with bDMARDs on TB rates. The availability of the earlier data was restricted by the existing law not allowing to get the data previous to 2012.

## 7. Conclusions

This study is the first nationwide cohort study to assess the incidence of TB in a broad spectrum of chronic inflammatory RD. The incidence of TB in RD cohort was highly consistent with that predicted by national estimates, with a resultant SIR of 0.90 (0.41–1.70). The risk of TB by estimating HR in patients using bDMARDs versus nonusers was calculated to be 4.54 (0.94; 21.87) in a total RD cohort, although not statistically significant. Though limited by short follow-up period, this study may help to support the necessity of current TB screening strategy prior to immunosuppressive treatments. The results of our study indicate the need for future follow-up of the cohort of RD patients.

## Figures and Tables

**Table 1:** Patient characteristics.

Characteristic	No. TB	TB	Total
No. of patients	8770	9	8779
Rheumatoid arthritis (M05, M06)	4618 (53%)	5 (56%)	4623 (53%)
Spondyloarthropathies (M07, M45, M46)	2476 (28%)	3 (33%)	2479 (28%)
Systemic connective tissue diseases (M30-35)	1676 (19%)	1 (11%)	1677 (19%)
Female	6130 (70%)	5 (56%)	6135 (70%)
Mean age when rheumatic disease was diagnosed (min-max, SD)	56 (18–97, 16)	44 (18–66, 17)	56 (18–97, 16)
Mean years of follow-up (SD)	2.71 (1.43)	1.86 (0.45)	2.71 (1.43)
Total person months of follow-up	285,397, 98	200, 72	285,598, 70
No. of patients with biologicals	456 (5%)	2 (22%)	458 (5%)

TB: Tuberculosis; SD: standard deviation.

**Table 2:** Incident cases of tuberculosis and standardized incidence ratios.

	Incident Cases of Tuberculosis	Person Years of Follow-Up in a Total Cohort	Rate per 100,000 Person Years (95% CI)	Expected Incident TB Cases from Official Statistics Website 2013–2017	Standardized Incidence Ratio (95% CI)
Rheumatoid arthritis (M05, M06)	5	12,822.55	38.99 (14.29;86.43)	4.7	1.05 (0.34;2.46)
Spondyloarthropathies (M07, M45, M46)	3	6639.98	45.18 (11.49;123.0)	3.8	0.80 (0.17;2.34)
Systemic connective tissue diseases (M30-35)	1	4337.37	23.05 (1.15;113.7)	1.5	0.66 (0.02;3.69)
All patients	9	23,799.90	37.81 (18.44;69.4)	10.0	0.90 (0.41;1.70)
Female	5	16,826.46	29.71 (10.89;65.86)	4.1	1.21 (0.39;2.81)
Male	4	6973.43	57.36 (18.23;138.4)	5.9	0.68 (0.19;1.75)

CI: confidence interval.

**Table 3:** Tuberculosis risk evaluation for exposed versus unexposed to bDMARDs in total rheumatic diseases (RD) cohort and in rheumatoid arthritis patients.

Total RD Cohort (*n* = 8779)	Hazard ratio (95% CI)
bDMARD use versus no bDMARD use (unadjusted)	4.54 (0.94; 21.87)
bDMARD use versus no bDMARD use (adjusted for age and gender)	3.53 (0.71; 17.49)
**Rheumatoid arthritis patients (*n* = 4623)**	**Hazard ratio (95% CI)**
bDMARD use versus no bDMARD use (unadjusted)	4.55 (0.51; 40.70)
bDMARD use versus no bDMARD use (adjusted for age and gender)	3.44 (0.37; 32.16)
